# From gaming to surgery: the influence of digital natives on robotic skills development

**DOI:** 10.1007/s11701-024-02178-0

**Published:** 2024-11-29

**Authors:** Dominik Imre Szabó, András Vereczkei, András Papp

**Affiliations:** Department of Surgery, Clinical Centre, Medical School, University of Pécs, Pécs, Hungary

**Keywords:** Da Vinci Surgical System, Robotic surgery, Training, Video game usage, Age

## Abstract

Our study investigates the potential correlation between generational differences, like age and previous experience with digital innovations, such as video games and smartphones, and the performance on the Da Vinci Skills Simulator, the cornerstone of robotic surgery training. Thirty participants were involved from three age groups: Generation Alpha, Generation Z, and Generation X. None had prior robotic surgical experience. Participants performed the Wrist Articulation 1 task on the Da Vinci Skills Simulator after two practice rounds. Analysis of performance metrics and statistical tests were conducted to assess the differences between groups. Additionally, participants had completed a survey on their habits related to video gaming, smartphone, and computer usage. A trend was observed where performance declined with age, meaning that Generation Alpha performed the most successfully compared to the other generations, although the difference was not statistically significant (*p* = 0.51). However, significant differences were found in Glass Movement (GM) by Generation Z showing superior precision, making less errors (*p* = 0.019). The study found no correlation between simulator performance and early or frequent exposure to smartphones or video games. Interestingly, frequent two-thumb typing on smartphones correlated with better performance in the GM metric (*p* = 0.006). Generation Z demonstrated greater precision in handling the simulator, reflecting that robotic surgery training might be best to be started at the beginning of residency programs. Young doctors using two-thumb typing develop robotic surgery skills faster. Further studies are needed to determine whether this quicker learning would also lead to better skills later on.

## Introduction

The last decades have seen a significant ascent in minimally invasive surgery with the spread of laparoscopic surgery (LS) and more recently robot-assisted surgery (RAS) [1]. Robotic surgical systems provide features like 10x magnification, tremor filter, a three-dimensional view, and a more ergonomic stance for the operating surgeon, allowing a more precise dissection, a decrease in blood loss, and faster recovery [2–4]. The Da Vinci Skills Simulator is a computer-based virtual-reality system that grants to practice both basic maneuvers and more advanced techniques for robotic surgeon aspirants. Even simulated surgeries are available to be performed. It was found to be the most cited simulator when looking at published robotic surgery curricula [5]. Compared to other virtual-reality robotic simulators, the Da Vinci Skills Simulator was found to have the highest score in face and content validity [6]. Furthermore, relationship has been investigated between simulator and clinical robotic performance, meaning that skills seem to be transferable [7, 8].

Several studies aimed to find non-surgical factors associated with a more successful result on the simulator. Widely researched determinants are video gaming history, IQ, and dexterity [9]. Prior laparoscopic experience has been suggested to have the strongest impact [10]. With the understanding of these variants, the training of robotic surgeons may undergo a revision, as studies have suggested that simulators are essential in training protocols. Additionally, distinguishing between surgeon aspirants, the revealing of innate abilities may become more straightforward [11]. A study with limited participants previously proposed that age may be such a factor, as the younger generations are born into an environment where digital innovations are used in everyday life [12]. Hand–eye coordination, dexterity, and conversion of two-dimensional screen image into three-dimensional perception are requirements for both video gaming, smartphone use, and also performing minimally invasive surgery. Therefore, acquiring these skills at a young age may lead to a quicker and more precise use of the surgical system. Our goal was to determine whether the age of naïve individuals, and video gaming or smartphone experience would influence the adaption to the controls of the Da Vinci Surgical System.

## Methods

### Participants

Thirty individuals were evenly divided into three groups by age, such as Children (age: 13–15 years), Young adults (age: 23–27 years), and Adults (age: 45–68 years). The selected age groups represented Generation Alpha, Generation Z, and Generation X.

### Simulator testing

After instructing the participant on finger positioning, the use of the clutch switch, and other controls, all subjects were asked to perform on the Da Vinci Skills Simulator. The selected exercise is considered as one of the simplest, since all of the subjects were unprofessional in the field of robotic surgery and children were also included, the task was appointed accordingly. Namely, Wrist Articulation 1 only requires basic hand–eye coordination, depth perception, and dexterity. The exercise expects the controller to move the instruments through a hole on a glass ball to reach the target inside. This must be completed ten times, involving both the left hand and right hand, with the hole positioned in various locations. The participants had two rounds of practice, before the results of the third attempt were documented. The scoring system had been briefly explained and a tutorial of the required task had been demonstrated. The Da Vinci Skills Simulator provides a detailed summary following each attempt, containing: Overall Score (OS), Total Time (TT), Economy of Motion (EM), Penalty (P), Glass Movement (GM), Instrument Collision (IC), Success Rate (SR), and Number of Times Instrument out of View (TOV) (Table 1). These variables were the basis of the statistical analysis; however, the latter three metrics were excluded, since all individuals performed the best score.

**Table 1:** Descriptions of performance metrics provided by Skills Simulator after completing Wrist Articulation 1

Metric	Description
Overall Score (OS)	A value given from 0 to 100 based on Total Time, Economy of Motion, and the penalties calculated by the algorithm of Intuitive
Total Time (TT) (s)	The time spent completing the task, the lower is better
Economy of Motion (EM) (cm)	The distance that the instruments traveled during the task. The lower is better, meaning no unnecessary movement was made
Penalty (P)	A summed and rounded value of the penalty metrics: Glass Movement, Instrument Collision, Success Rate, Number of Times Instrument is out of View
Glass Movement (GM) (cm)	While reaching for the target, the movement of the outer glass sphere. The lower is better
Instrument Collision (IC) (ct.)	Number of times the instruments were collided during the task. The lower score is better
Success rate (SR) (%)	Representing how many of the ten positions were successfully completed. The higher is better
Number of Times Instruments are out of View (TOV) (ct.)	Representing how many times the instruments were moved out of view. The lower is better

### Questionnaire

In addition to completing an exercise on the Da Vinci Skills Simulator, a survey was conducted including 18 questions. First, data were collected about age, occupation, education, and hand dominance. Then, multiple questions meant to explore the subjects’ habits regarding video gaming experience, computer, and smartphone usage. Age at first meeting these IT devices and current frequency of utilization were in focus.

### Data analysis

The purpose was to statistically prove a significant difference between the age groups’ results, and also between the performances of other groups based on the answers given on the survey. Consequently, ANOVA was carried out, since we had three or more groups to compare. Therefore, assumption checks had had to be made. For inspecting the normality of the variables, Shapiro–Wilk test had been used and Levene’s test had been used for variance analysis. When normality examinations failed, Kruskal–Wallis non-parametric test was applied. Welch’s test was used in case the variances were not homogenous. Data analysis was done using Jamovi (Sydney, Australia) for Windows, version 2.5.6. Statistical significance was set at *p* values <0.05.

## Results

### Demographics

Overall, 30 participants were enrolled in the study, 10 children, 10 young adults, and 10 adults. All of them completed both the questionnaire and the task on the simulator. One subject was excluded due to lack of sufficient cooperation, leaving the Generation Alpha group with nine participants.

The Children group included males only; all of the subjects were aged 13, 90.0% was right-handed. Mean age of the young adult participants was 24 ± 1.3; the majority were female (70.0–30.0%). In contrast to the youngsters, all of them were right-handed. In the Adult group, 50.5 ± 7.1 was the mean age, only right-handed participants were included and a female dominance was seen among the subjects (60.0%) (Table 2). All 30 participants came from the middle class, as the participants were mostly university graduates or currently studying in higher education institutes or members of a white-collar family.

**Table 2:** Demographics of participants

	Age group		
	Children (Generation Alpha)	Young adults (Generation Z)	Adults (Generation X)
N	9	10	10
Sex			
Female	0 (0.0%)	7 (70.0%)	6 (60.0%)
Male	9 (100.0%)	3 (30.0%)	4 (40.0%)
Mean of age	13 ± 0.0	24 ± 1.3	50.5 ± 7.1
Handedness	8 (88.9%) right-handed	10 (100.0%) right-handed	10 (100.0%) right-handed

### Outcomes of questionnaire and simulator performance

Analysis of the survey revealed that younger generations got in contact with technical innovations, video games, and smartphones, at a much younger age. From the Generation Alpha group, eight out of nine (88.8%) children first used smartphones and first played video games before the age of 10. When asked about current usage, six out of nine (66.6%) responded that they play video games very often/often. In addition, seven (77.7%) children replied that they had experienced virtual reality a few times or several times. Compared to this, responses on the same questions from Generation Z revealed that only five out of ten people (50.0%) first played video games before the age of 10. These participants acquired smartphones only between the age of 10 and 15. Presently, three out of ten (30%) young adults play video games often. Looking at Generation X, nine out of ten (90.0%) said that they first used smartphones after the age of 20; furthermore, six out of those nine people answered that they were even older than 30. None of the participants in this group played video games before the age of 10. Four out of ten (40.0%) subjects said that they tried video games between the age of 10 and 15; equivalent number said that they first played those only after the age of 20, making the curve bimodal.

When asked about typing with two thumbs on smartphones, 15 of the 29 (51.7%) participants said that it was the method they use exclusively.

**Fig. 1:**
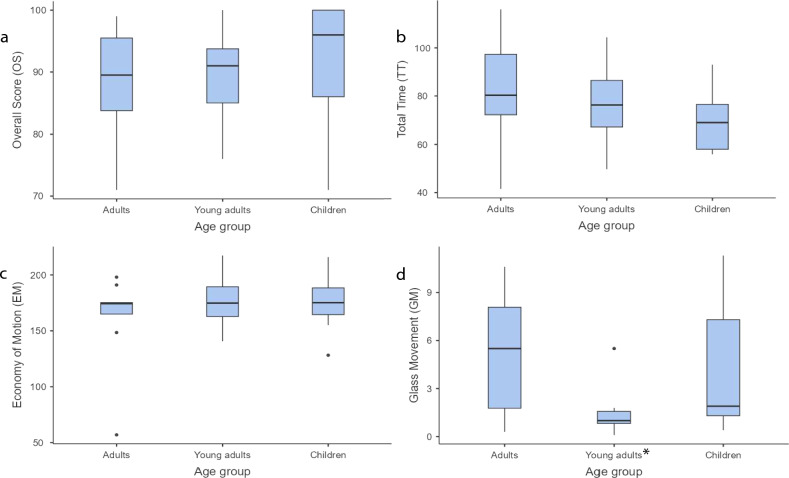
Comparison of results achieved by age groups. Overall Score **a**, Total Time (s) **b**, and Economy of Motion (cm) **c** were similar among Children, Young adults, and Adults. Glass Movement (cm) **d** was significantly less in case of Young adults (*p = 0,019)

Results on the Da Vinci Skills Simulator revealed a trend. Overall Scores (OS) gradually declined as the age increased; means of the groups were 91.7 ± 9.9, 89.8 ± 7.6, and 87.7 ± 9.8. The difference is conspicuous, however, not statistically significant (*p* = 0.51). Total time (TT) followed a similar tendency, children’s mean was 69.7 ± 12.1, young adults’ 76.6 ± 16.1, and the mean of adults’ was 81.6 ± 21.9. Glass Movement (GM) showed interesting results, Generation Z performed a surprisingly low mean (1.47 ± 1.5) compared to the others (Children: 4.05 ± 3.8, Adults: 5.2 ± 3.6), making this difference significant (*p* = 0.019) (Table 3, Fig. 1).

**Table 3:** Comparison of results achieved by age groups

	Age group	Overall score (OS)	Total time (TT)	Economy of motion (EM)	Glass movement (GM)
N	Adults	10	10	10	10
	Young adults	10	10	10	10
	Children	9	9	9	9
Mean	Adults	87.700	81.660	162.360	5.210
	Young adults	89.800	76.630	177.120	1.470 *
	Children	91.778	69.700	173.178	4.056
Standard deviation	Adults	9.787	21.904	39.456	3.661
	Young adults	7.671	16.186	22.431	1.506
	Children	9.909	12.107	24.509	3.804

Overall Score, Total Time (s), and Economy of Motion (cm) were similar among Children, Young adults, and Adults. Glass Movement (cm) was significantly less in case of Young adults (*p = 0,019)

**Fig. 2:**
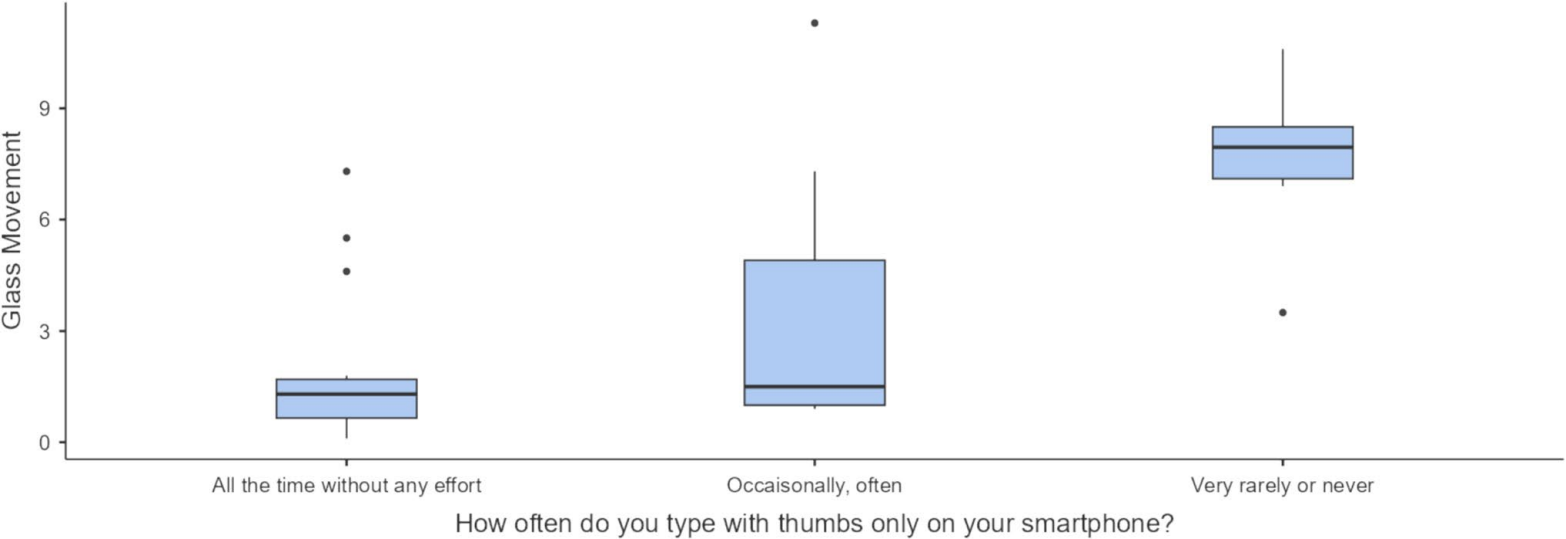
Those who never or very rarely type on their smartphones using their two thumbs performed poorer in Glass Movement (cm) metric (*p = 0.006)

Regarding genders, there was no difference between the results of males and females; both completed the task with the same outcome (OS *p* = 0.76). A link was not established between a successful score and how early smartphones (*p* = 0.57) and video games (*p* = 0.33) were first used and played. Our study was not able to confirm the association between performance on the Da Vinci Skills Simulator and the frequency of playing video games (*p* = 0.175). However, our results suggest that Glass Movement (GM) depends on how often and quickly both of the thumbs are used for typing on smartphones, those who never or very rarely do it performed significantly poorer in this metric (*p* = 0.006) (Table 4 Fig. 2).

**Table 4:** Statistical results of groups, based on their response in the following question, representing a difference in their performance in one of the penalty metrics

	How often do you type with your two thumbs only on your smartphone?	Glass movement
N	All the time without any effort	15
	Occasionally, often	8
	Very rarely or never	6
Mean	All the time without any effort	1.947
	Occasionally, often	3.575
	Very rarely or never	7.583
Standard deviation	All the time without any effort	2.119
	Occasionally, often	3.839
	Very rarely or never	2.352

## Discussion

Twenty-three years after the transatlantic robot-assisted telesurgery executed by Jacques Marescaux, the Da Vinci Surgical System has spread all over the globe with more than 9000 units installed [13]. Between 2012 and 2018, an increase of 13.3% was documented in the use of robotic surgery [1]. In 2023, the Da Vinci Surgical System was used in more than 2.2 million procedures, making the total count over 14 million [14]. Along with the benefits provided to the surgeon while operating, such as tremor filter, three-dimensional view, and more degrees of freedom, patient advantages have been circumscribed as well. Studies were published from various fields of surgery with the conclusion that compared to laparoscopic surgery, the risk for open conversion is lower besides a shorter length of hospitalization. Additional improvements were noticed in the rate of intraoperative complications and blood loss with the downside of longer operation time and higher costs [15–18]. These findings propose that robot-assisted surgery has a place among gold standard procedures in the future. Many publications and reviews have concluded that robot-assisted surgery should be regarded as an alternative to laparoscopic surgery [19, 20]. Even when studies did not find significant advantages from the aspect of the patients and stated that the outcome is equivalent, improvements in demands from the surgeon were still present [3, 4]. In spite of the prominent conquest of robotic surgery, many unknowns are still present; a worldwide elaborate evidence-based training protocol is lacking. A previous study found that 60% of general surgery residents participate in their first robotic case without prior education or training; however, programs had been introduced with the hopes of standardization [21, 22]. A review examining 39 robotic surgery curricula revealed severe heterogeneity and deficiencies. It suggested the use of educational frameworks to address these issues [23]. In 2024, a protocol was developed to achieve a pan-European consensus on the essential components of a common training program for GI robotic surgery [24]. One of its pillars is simulation education. The same year’s European Society of Coloproctology guideline on training in robotic colorectal surgery states that simulation should be used as part of training curriculum as strong recommendation [25].

Many publications delve into various factors playing a role in performing with the Da Vinci Skills Simulator, most importantly, devoted to establishing a link with video game experience [26, 27]. While other studies have discovered surprising determinants like playing sports or playing a musical instrument [28]. Our study was designed to explore the topic with a more holistic approach primarily, and looking at exact factors secondly. We investigated how different generations with total lack of experience in robotic systems and laparoscopic practice can adjust to controlling the simulator relying purely on the skillset gathered from other origins. Since the simulator operates with a computer-based virtual-reality interface, and with the assumption that youngsters nowadays grow up in an IT rich environment, we hoped to conclude that their performance will be more successful compared to the older generations. In our study, those among the children were selected, who already presented adequate commitment to the task and were able to control the simulator responsibly. The questionnaire served as a proof, that today’s children, Generation Alpha, get in contact with smartphones, video games at a much younger age along with a greater frequency in usage. Our study included children from the middle class mainly; however, we feel confident that our observations are true for the whole generation, alike habits are maintained by the whole society.

Following two practice rounds on the designated task, the results of the third were the basis of statistical analysis. The difference among the generations did not prove to be statistically significant; however, the means displayed the trend we had hoped for. Generation Alpha had the best result in Overall Score and Total Time. Additionally, we witnessed during the practice rounds that youngsters immediately achieved near excellent scores, and fine corrections were only needed for a perfect result. On the other hand, adults had a much harder time getting comfortable with the controls and performed poorly on the first tries. Thus, investigating learning curves could be a promising extension of the study, seeing that previous studies with limited participants suggested that the learning curve is not affected by the mentioned factors only the starting point varies [12].

The key finding was that young adults, participants from Generation Z, are more careful, precise when performing on the simulator, considering that how few penalty points from Glass Movement metric were gathered by them (Table 3 Fig. 1). The missing haptic feedback from current robotic surgical systems was handled the most successfully by them. People in their 20s present adequate vigilance and commitment to the task, and were born into an environment stacked with technology and electronic devices in contrast to the others, who may lack some of the above stated. The new Da Vinci 5 platform equipped with haptic feedback may level the difference reported between age groups [29].

Furthermore, a surprising finding was that typing on smartphones with two fingers fluently rather than using only one may be a skill that enables a more successful simulator experience (Table 4 Fig. 2).

Limitations of this study have to be noted. Only 30 participants were included with one exclusion, leaving a need for more and making it hard to identify significant differences in the results. Besides quantity, the young adult group mainly consisted of medical students, making the group less diverse. Overall ratio of genders was balanced; however, within the groups, major disproportions were witnessed, only male children were involved, and female dominance was present in the young adult group. In addition, only one task was required and no data were registered during the practice rounds. Perhaps, applying more exercises, even harder ones, would result in more significant differences in the outcome.

This study is unique, as we investigated performance on the Da Vinci Skills Simulator with a novel approach. To our knowledge, this is the first study that was conducted with participants aged between 13 and 15, naïve to robotic surgical systems. We demonstrated how generations differ, especially in the aspect of errors made. The results exhibit a need for a more comprehensive study welcoming a larger number of participants preferably necessitating more than one exercise. It also suggests that a possible extension to the topic may be the investigation of learning curves in a similar setup.

## Conclusion

Our study suggests that Generation Alpha may have a head start in controlling the robotic systems, considering they achieved the best score in both Overall Score and Total Time metrics. Moreover, it showed that today’s young adults in their 20s are performing with fewer mistakes on the Da Vinci Skills Simulator. We also revealed a surprising connection; typing on smartphones may contribute to a better result on the simulator. However, meeting the IT innovations of video games, smartphones earlier in life, and the frequency of their utilization was not found to be a determinant of simulator performance. More research is necessary to identify the factors playing a role in the ability of controlling robotic systems, thus promoting robotic surgery.

## Data Availability

No datasets were generated or analyzed during the current study.

## References

[1] Sheetz K, Claflin J, Dimick J (2020) Trends in the adoption of robotic surgery for common surgical procedures. JAMA Netw Open 25:e1918911. 10.1001/jamanetworkopen.2019.18911 10.1001/jamanetworkopen.2019.18911 PMC6991252 31922557

[2] Yeung T, Larkins K, Warrier S, Heriot A (2024) The rise of robotic colorectal surgery: better for patients and better for surgeons. J Robot Surg 18:69. 10.1007/s11701-024-01822-z 38329595 10.1007/s11701-024-01822-z

[3] Shugaba A, Subar D, Slade K et al (2023) Surgical stress: the muscle and cognitive demands of robotic and laparoscopic surgery. Ann Surg Open 4:e284. 10.1097/as9.0000000000000284 37342254 10.1097/AS9.0000000000000284 PMC7614670

[4] Shugaba A, Lambert J, Bampouras T, Nuttall H, Gaffney C, Subar D (2022) Should all minimal access surgery be robot-assisted? a systematic review into the musculoskeletal and cognitive demands of laparoscopic and robot-assisted laparoscopic surgery. J Gastrointest Surg 26:1520–1530. 10.1007/s11605-022-05319-8 35426034 10.1007/s11605-022-05319-8 PMC9296389

[5] Chen R, Rodrigues Armijo P, Krause C, Siu K, Oleynikov D (2020) A comprehensive review of robotic surgery curriculum and training for residents, fellows, and postgraduate surgical education. Surg Endosc 34:361–367. 10.1007/s00464-019-06775-1 30953199 10.1007/s00464-019-06775-1

[6] Hertz A, George E, Vaccaro C, Brand T (2018) Head-to-head comparison of three virtual-reality robotic surgery simulators. JSLS 22:e201700081. 10.4293/JSLS.2017.00081 10.4293/JSLS.2017.00081 PMC5863693 29618918

[7] Aghazadeh M, Mercado M, Pan M, Miles B, Goh A (2016) Performance of robotic simulated skills tasks is positively associated with clinical robotic surgical performance. BJU Int 118:475–481. 10.1111/bju.13511 27104883 10.1111/bju.13511

[8] Schmidt M, Köppinger K, Fan C et al (2021) Virtual reality simulation in robot-assisted surgery: meta-analysis of skill transfer and predictability of skill. BJS Open 5:zraa066. 10.1093/bjsopen/zraa066 33864069 10.1093/bjsopen/zraa066 PMC8052560

[9] Hagen M, Wagner O, Inan I, Morel P (2009) Impact of IQ, computer-gaming skills, general dexterity, and laparoscopic experience on performance with the da Vinci ® surgical system. Int J Med Robot Comput Assist Surg 5:327–331. 10.1002/rcs.264 10.1002/rcs.264 19455549

[10] Davila D, Helm M, Frelich M, Gould J, Goldblatt M (2018) Robotic skills can be aided by laparoscopic training. Surg Endosc 32:2683–2688. 10.1007/s00464-017-5963-5 29214515 10.1007/s00464-017-5963-5

[11] Moglia A, Ferrari V, Morelli L et al (2014) Distribution of innate ability for surgery amongst medical students assessed by an advanced virtual reality surgical simulator. Surg Endosc 28:1830–1837. 10.1007/s00464-013-3393-6 24442679 10.1007/s00464-013-3393-6

[12] Meier M, Horton K, John H (2016) Da Vinci© Skills Simulator™: is an early selection of talented console surgeons possible? J Robot Surg 10:289–296. 10.1007/s11701-016-0616-6 27334771 10.1007/s11701-016-0616-6

[13] Marescaux J, Leroy J, Gagner M et al (2001) Transatlantic robot-assisted telesurgery. Nature 413:379–380. 10.1038/35096636 11574874 10.1038/35096636

[14] “Intuitive Surgical, Annual Report 2023.” https://isrg.intuitive.com/static-files/e9ed7c14-f042-4923-b7cc-a1458e11e67b. Accessed 21 Sep 2024

[15] Choi J, Diab A, Tsay K et al (2024) The evidence behind robot-assisted abdominopelvic surgery: a meta-analysis of randomized controlled trials. Surg Endosc 38:2371–2382. 10.1007/s00464-024-10773-3 38528261 10.1007/s00464-024-10773-3

[16] Safiejko K, Tarkowski R, Koselak M et al (2021) Robotic-assisted vs. standard laparoscopic surgery for rectal cancer resection: a systematic review and meta-analysis of 19,731 patients. Cancers (Basel) 14:180. 10.3390/cancers14010180 35008344 10.3390/cancers14010180 PMC8750860

[17] Guerrini G, Esposito G, Magistri P et al (2020) Robotic versus laparoscopic gastrectomy for gastric cancer: The largest meta-analysis. Int J Surg 82:210–228. 10.1016/j.ijsu.2020.07.053 32800976 10.1016/j.ijsu.2020.07.053

[18] Wang L, Zeng W, Wu Y, Gong Z (2024) Comparison of clinical efficacy and safety between robotic-assisted and laparoscopic adrenalectomy for pheochromocytoma: a systematic review and meta-analysis. J Robot Surg 18:115. 10.1007/s11701-024-01846-5 38466492 10.1007/s11701-024-01846-5

[19] Ataya K, Bsat A, Aljaafreh A, Bourji H, Al Ayoubi A, Hassan N (2023) Robot-assisted heller myotomy versus laparoscopic heller myotomy: a systematic review and meta-analysis. Cureus 15:e48495. 10.7759/cureus.48495 38073943 10.7759/cureus.48495 PMC10704850

[20] Ziogas I, Giannis D, Esagian S, Economopoulos K, Tohme S, Geller D (2021) Laparoscopic versus robotic major hepatectomy: a systematic review and meta-analysis. Surg Endosc 35:524–535. 10.1007/s00464-020-08008-2 32989544 10.1007/s00464-020-08008-2

[21] Tom C, Maciel J, Korn A et al (2019) A survey of robotic surgery training curricula in general surgery residency programs: how close are we to a standardized curriculum? Am J Surg 217:256–260. 10.1016/j.amjsurg.2018.11.006 30518480 10.1016/j.amjsurg.2018.11.006

[22] Moit H, Dwyer A, De Sutter M, Heinzel S, Crawford D (2019) A standardized robotic training curriculum in a general surgery program. JSLS 23:e2019 00045. 10.4293/JSLS.2019.00045 31892790 10.4293/JSLS.2019.00045 PMC6924504

[23] Rahimi A, Ho K, Chang M et al (2023) A systematic review of robotic surgery curricula using a contemporary educational framework. Surg Endosc 37:2833–2841. 10.1007/s00464-022-09788-5 36481821 10.1007/s00464-022-09788-5

[24] Fadel M, Walshaw J, Pecchini F et al (2024) European Robotic Surgery Consensus (ERSC): Protocol for the development of a consensus in robotic training for gastrointestinal surgery trainees. PLoS ONE 19:e0302648. 10.1371/journal.pone.0302648 38820412 10.1371/journal.pone.0302648 PMC11142498

[25] Tou S, Au S, Clancy C et al (2024) European Society of Coloproctology guideline on training in robotic colorectal surgery (2024). Colorectal Dis 26:776–801. 10.1111/codi.16904 38429251 10.1111/codi.16904 PMC12150670

[26] Harbin A, Nadhan K, Mooney J et al (2017) Prior video game utilization is associated with improved performance on a robotic skills simulator. J Robot Surg 11:317–324. 10.1007/s11701-016-0657-x 27853947 10.1007/s11701-016-0657-x

[27] Kılınçarslan Ö, Türk Y, Vargör A, Özdemir M, Hassoy H, Makay Ö (2023) Video gaming improves robotic surgery simulator success: a multi-clinic study on robotic skills. J Robot Surg 17:1435–1442. 10.1007/s11701-023-01540-y 36754922 10.1007/s11701-023-01540-y

[28] Harper J, Kaiser S, Ebrahimi K et al (2007) Prior video game exposure does not enhance robotic surgical performance. J Endourol 21:1207–1210. 10.1089/end.2007.9905 17949327 10.1089/end.2007.9905

[29] “Intuitive Surgical, Da Vinci 5. https://www.intuitive.com/en-us/products-and-services/da-vinci/5. Accessed 21 Sep 2024

